# Tear-derived extracellular vesicles as diagnostic biomarkers for ocular and neurodegenerative diseases: opportunities and challenges

**DOI:** 10.20517/evcna.2025.72

**Published:** 2025-09-30

**Authors:** Marta Sanroque-Muñoz, Sergio G. Garcia, Linrong Pan, Marta Clos-Sansalvador, Miriam Font-Morón, Jéssica Botella-Garcia, Jordi Loscos-Arenas, Francesc E. Borràs

**Affiliations:** ^1^REMAR-IGTP Group, Health Science Research Institute Germans Trias i Pujol (IGTP), Can Ruti Campus, Badalona 08916, Spain.; ^2^Department of Biochemistry and Molecular Biology, Universitat Autònoma de Barcelona (UAB), Bellaterra 08913, Spain.; ^3^Department of Cell Biology, Physiology, and Immunology, Universitat Autònoma de Barcelona (UAB), Bellaterra 08913, Spain.; ^4^Ophthalmology Service, Hospital Universitari Germans Trias i Pujol, Badalona 08916, Spain.; ^5^Department of Cell Biology, Physiology, and Immunology, Universitat de Barcelona (UB), Barcelona 08028, Spain.

**Keywords:** Ocular diseases, neurodegenerative diseases, extracellular vesicles, tears, biomarkers

## Abstract

In recent years, the prevalence of ocular diseases has increased considerably. However, timely diagnosis and treatment are hampered by the challenge of early detection since symptoms often appear in advanced stages. Emerging research highlights extracellular vesicles (EVs) as potential biomarkers for ocular diseases, with tear-derived EVs offering a minimally invasive source for early diagnosis. Tears play a crucial role in maintaining eye health and reflect the physiological state of the eye; thus, abnormalities in tear composition can provide valuable insight into inflammatory eye diseases. Studies have demonstrated the utility of tear-derived EVs in identifying biomarkers not only for inflammatory eye diseases but also for neurodegenerative disorders, as they carry molecular signatures (including proteins and various RNA species) reflective of their cells of origin. In this review, we discuss the potential of tear-derived EVs as biomarkers for early detection and monitoring of ocular and neurodegenerative diseases and highlight the importance of standardizing tear collection and EV isolation protocols to ensure reproducibility.

## INTRODUCTION

The incidence of ocular diseases has increased significantly over the past decade, affecting at least 2.2 billion individuals worldwide^[1]^. These conditions are particularly prevalent among middle-aged and elderly populations, causing vision impairment and, in many cases, irreversible blindness^[2]^. A key challenge in addressing ocular diseases is the delayed onset of symptoms and the difficulty in obtaining biopsy samples, which hampers early diagnosis and treatment. Conventional diagnostic methods require baseline assessments and, therefore, often detect only advanced or end-stage disease^[3,4]^. Moreover, many ocular disorders, such as glaucoma, age-related macular degeneration (AMD), and diabetic retinopathy, often remain asymptomatic until significant and irreversible damage has already occurred^[4-6]^. This silent progression limits the opportunity for clinicians to intervene while vision could still be preserved. Current diagnostic approaches, including fundoscopy, visual field testing, and optical coherence tomography (OCT), while highly valuable, have their own limitations^[7,8]^. Their sensitivity for detecting early molecular alterations remains low, and results can be influenced by operator expertise, patient compliance, and the availability of specialized equipment, which is not always accessible^[9]^. These constraints highlight the need for novel screening strategies that enable early detection of sight-threatening conditions and more effective monitoring of disease progression.

Extracellular vesicles (EVs) - cell-derived membranous structures - have been extensively studied for their role in mediating a plethora of intercellular activities. They are involved in numerous physiological and pathological processes, and EV-related molecules have shown considerable promise as novel biomarkers in several diseases in the last decade^[10]^.

Within this field, tear fluid has emerged as an attractive source of EVs. Unlike blood or serum, which are highly viscous and contain large amounts of proteins and lipids that could hinder EV isolation^[11]^, tears are easier to process and yield molecules at relatively high concentration and purity^[12]^. Studies have shown significant molecular overlap between plasma and tears, including shared proteins and microRNAs, while also identifying molecules unique to tears^[13]^. Notably, tear fluid not only reflects systemic alterations but also contains EVs derived directly from ocular cells, providing disease-specific molecular signatures of particular relevance for eye disorders^[14]^.

In this review, we summarize the most recent literature on the use of EVs for the detection of ocular diseases, with a special focus on tear-derived extracellular vesicles as a minimally invasive source of biomarkers, including their potential relevance in neurodegenerative disorders.

## EYE STRUCTURE AND OCULAR FLUIDS AS A SOURCE OF BIOMARKERS

The eye is a bilateral and spherical organ that houses the structures responsible for vision. It is situated within the orbit and consists of the eyeball and its accessory structures. The eyeball itself is composed of three layers^[15]^. The outer layer comprises the cornea, the transparent part of the eye that allows light to enter, and the sclera, the white part of the eye. The middle layer is the choroid, whose anterior portion is the iris, the colored part of the eye that contains the pupil, a circular aperture regulating the amount of light entering the eye. The inner layer consists of the retina and includes the optic nerve, which transfers visual information to the brain. Surrounding the eyeball lies the conjunctiva, a membrane that covers the sclera and lines the inner eyelids. The conjunctiva and the cornea are continuously bathed by the tear film, which is essential for the production and distribution of tears, thereby maintaining ocular lubrication and protection. The interior of the eyeball is filled with aqueous humor and vitreous humor, which, together with the tears, support the vitality of tissues and provide lubrication, nourishment, and protection essential for vision^[16]^.

Vitreous humor (VH) is a transparent gel-like extracellular matrix composed mainly of water and a meshwork of fine collagen fibrils, hyaluronan molecules, lipids, and inorganic salts^[17,18]^. It fills the space between the lens and the retina (posterior chamber), providing shape, elasticity, and volume, and facilitating light transmission to the retina^[19]^. VH captures proteins that are either secreted locally by the retina or diffused from adjacent ocular tissues, which positions it as a valuable source for investigating biomarkers associated with vitreoretinal diseases, including diabetic retinopathy and age-related macular degeneration^[20,21]^.

Aqueous humor (AH) is a clear fluid located in the anterior chamber of the eye, between the cornea and the lens. Although predominantly water, it also contains small quantities of sugars, proteins, vitamins, cytokines, growth factors, and other nutrients^[22]^. AH maintains intraocular pressure, nourishes the anterior segment of the eye, and removes metabolic waste^[3]^. It is secreted by the non-pigmented ciliary epithelium and drains through the trabecular meshwork or the uveoscleral pathway^[23]^. Accordingly, AH is considered an important source of biomarkers in glaucoma^[24,25]^. Given its interaction with vitreous humor, it also holds potential for biomarker discovery in retinal pathologies^[26,27]^.

**Figure 1:**
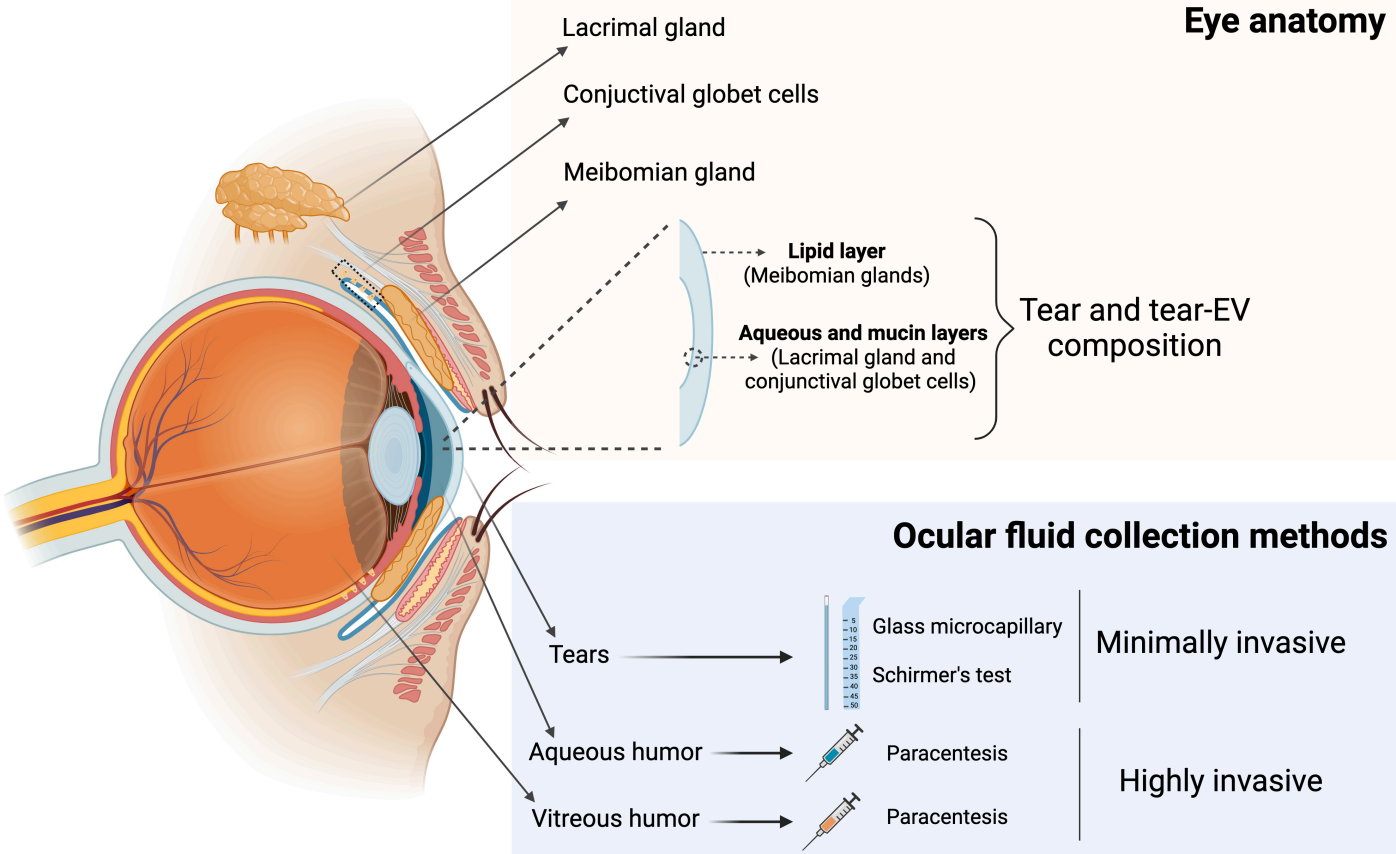
Schematic representation of an anatomic eye with its associated glands, the three-layer composition of tear film, and different ocular fluid collection methods. Created with BioRender. Borràs, F. (2025) https://BioRender.com/cpfv79z. EV: Extracellular vesicle.

The tear film is a transparent fluid that covers the ocular surface. It is composed of three layers: an outer lipid layer, a middle aqueous layer, and an inner mucin layer^[28]^. The meibomian glands secrete most of the lipids in the tear film lipid layer, while the lacrimal glands and conjunctival goblet cells are mainly responsible for producing the aqueous and mucin components of the tear film, respectively^[29]^ [Figure 1].

Tears play a crucial role in maintaining ocular health by providing hydration and lubrication to the mucosal surface, protecting against pathogens, clearing metabolic waste, and nourishing the underlying corneal and conjunctival epithelial cells^[30]^. They also serve as a valuable source of biomarkers for disorders of the anterior segment of the eye, such as dry eye syndrome and conjunctivitis^[31,32]^.

Ocular fluids, produced by ocular glands and cells and by surrounding tissues and vasculature^[16]^, may reflect the health and/or pathological state of the eye^[33]^. Moreover, ocular fluids are in direct contact with disease sites, which may be an additional advantage compared to detecting biomarkers in blood or plasma, where molecules may be highly diluted^[30]^.

However, the use of aqueous and vitreous humor for biomarker discovery is limited by the invasiveness of the collection process [Figure 1].

Aqueous humor is obtained via anterior chamber paracentesis, and vitreous humor via vitreous aspiration, both procedures carrying significant risks, such as retinal tear or detachment^[34]^. Moreover, such invasive procedures are not ethically applicable to control subjects. In contrast, tear collection is minimally invasive [Table 1].

**Table 1:** Summary of ocular fluid collection methodologies

**Ocular fluid**	**Methodology**	**Invasiveness**	**Difficulty**	**Patient discomfort**
**Vitreous humor**	Vitreous aspiration	High	High	High
**Aqueous humor**	Retinal surgery process	High	High	High
**Tears**	Glass microcapillary	Low	Medium	Medium
	Schirmer test	Low	Low	Low

Tears are typically collected using glass microcapillaries^[3]^ or the Schirmer type I tear test, which involves inserting a filter paper strip into the lower conjunctival fornix for 5 minutes^[35]^. Collection with microcapillaries requires specific skills from the operator (doctor or nurse), though it could be interrupted by blinking and usually yields a small sample volume. Schirmer test, by contrast, is simpler to perform and generally perceived by patients as significantly less invasive^[36]^. However, samples may be contaminated with epithelial cells from the sub-palpebral skin, a factor that should be carefully considered during analysis. Despite the specific advantages and limitations of each collection method, tear fluid stands out as a minimally invasive and promising source of EVs, holding significant potential for biomarker discovery and disease monitoring across a broad spectrum of conditions. For this reason, research on tears as a source of biomarkers has increased in recent years, surpassing interest in other ocular fluids. Accordingly, this review aims to summarize the current knowledge on tear-derived extracellular vesicles as a promising source of biomarkers for both ocular and neurodegenerative diseases.

## BRIEFING ON EXTRACELLULAR VESICLES

EVs are secreted by most cell types and are present in a wide range of body fluids, including ocular fluids, blood, urine, breast milk, cerebrospinal fluid, lymph, amniotic fluid, and saliva^[37]^. Their lipid bilayer membrane encapsulates and protects their molecular content from enzymatic degradation, preserving it as a source of both physiological and pathological information. This encapsulation also facilitates cargo transfer between cells, enabling intercellular communication^[38]^. The complex composition of EV cargo reflects the state of their cell of origin and can influence the function and phenotype of recipient cells. Moreover, it is reported that when a functional molecule is delivered by EVs, it may be more active than in its soluble form^[38]^.

EVs are a heterogeneous nanosized population ranging from 30 to 1,000 nm, with different biogenesis mechanisms. Historically, they have been categorized into exosomes (originating from endosomal multivesicular bodies) and microvesicles (formed by membrane budding of the plasma membrane). Alternatively, EVs can be classified as small (< 200 nm) or large (> 200 nm) based on size. Yet, despite their specific biogenesis mechanisms, overlapping sizes and densities and shared molecular markers among different EV populations make their precise identification and separation challenging. Thus, the International Society for Extracellular Vesicles (ISEV) recommends the use of the generic term “EVs” while acknowledging operational extensions that include non-vesicular particles often co‐isolated with EVs, as outlined in MISEV2023 guidelines^[39]^. The molecular composition of EVs is diverse, encompassing proteins, lipids, carbohydrates, and genetic material. These components may be embedded within the vesicle membrane, constitute the internal cargo, or correlate with surface corona^[40]^.

**Figure 2:**
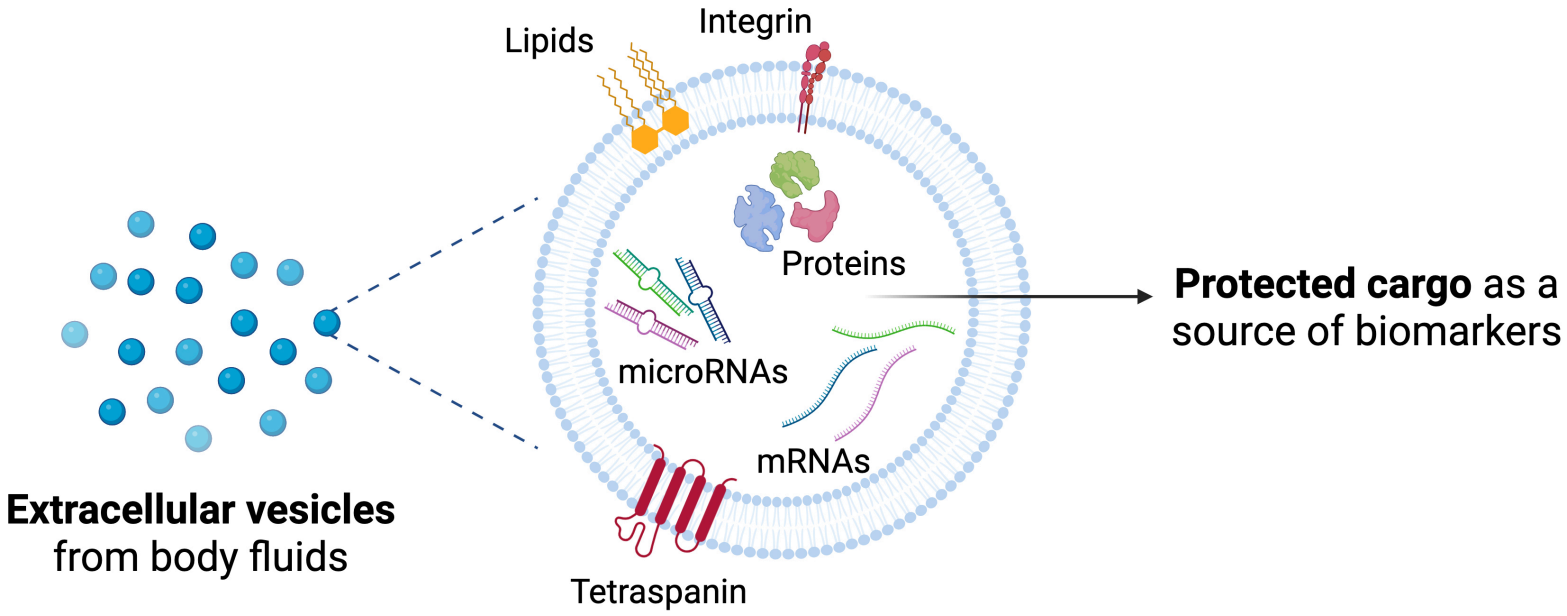
Extracellular vesicles as a source of biomarkers. Schematic view of protected cargo and membrane-associated molecules. Created in BioRender. Borràs, F. (2025) https://BioRender.com/cpfv79z.

Beyond universally expressed markers such as tetraspanins, EVs also carry cell-type-specific signatures that reflect cellular status and phenotype, along with their stability in biological fluids and the protection of their cargo [Figure 2].

Compared with whole cells, EVs offer enhanced stability, improved safety, increased tissue permeability, and lower tumorigenicity^[41]^. These properties allow EVs to cross biological barriers, such as the blood-brain and blood-retinal barriers, enabling the delivery of molecular information from different tissues^[42]^. These positioned EVs as promising candidates for defining potential biomarkers for the diagnosis and prognosis of various diseases^[39,43,44]^.

## TEAR AND TEAR-DERIVED EV COMPOSITION

The tear film has a volume of 3 to 10 μL and is secreted at a rate of 1 to 2 μL/min^[45]^. Its composition is primarily water (98,3%), with smaller fractions of salts (1%), proteins and glycoproteins (0.7%), and other minor constituents including metabolites^[46]^. Compared to blood and serum, tears contain significant amounts of proteins and other components, with lysozyme, lactoferrin, secretory IgA, and lipocalin being the four most abundant and well-characterized tear proteins^[47]^.

The biochemical composition of tears varies with context and stimulus type. Four distinct tear types have been identified: basal, reflex, closed-eye, and emotional tears. Basal tears are continuously produced under normal conditions to maintain ocular surface homeostasis. Reflex tears are secreted in response to irritants and in higher volumes than basal tears to help clear foreign substances from the ocular surface. Closed-eye tears are produced during sleep to ensure overnight lubrication of the eye, while emotional tears are associated with intense emotional states^[48]^. Protein, lipid, and secretory IgA concentrations differ among tear types, with basal tears containing the highest levels of protein and lipid content^[49]^. Nevertheless, compared to blood and serum, tears contain much lower amounts of proteins and other components^[50]^. Key tear film proteins, including lactoferrin, lipocalin-1, lysozyme, and tear-specific prealbumin^[47]^, remain relatively stable across the different tear types^[51]^, but are largely absent from serum^[52]^.

Tears also contain EVs, whose cargo content depends on the tear type. These EVs originate from the lacrimal glands, meibomian glands, goblet cells, and ocular surface epithelial cells^[53]^.

Abnormalities in tear film, affecting the constituents or the volume, can rapidly result in serious eye surface dysfunction and ultimately impair corneal transparency^[54]^. Disease-specific changes in tear proteins and metabolites have been identified in diseases such as dry eye syndrome, diabetic retinopathy, age-related macular degeneration, and glaucoma^[14,55-59]^. In diabetic retinopathy, for instance, inflammation and blood-retinal barrier dysfunction may alter retinal vasculature, increasing barrier permeability and reducing junction protein levels, which leads to a higher proportion of serum proteins in the tear film^[60]^. Due to their inherent ability to cross biological barriers, EVs from blood may also reach the retina, modifying ocular fluid composition. Consequently, tear composition, and specifically tear-derived EV content, could reflect the (patho)physiological state of the eye, providing specific information about the tissues underneath the eye and serving as a potential tool for evaluating ocular health and disease.

## TEAR COLLECTION AND TEAR-EV ISOLATION

In most studies using tears, the starting sample is reported as tear volume (µL) using a 10 µL capillary tube^[61,62]^, or as the tear fluid migration length (mm) on a Schirmer strip after 5 min of sampling, since the distance traveled by the tears is proportional to their production^[63]^. However, there is currently no standardized protocol for tear sample handling, from collection to analysis, particularly regarding storage format, duration, and temperature. All these parameters are important for subsequent EV analyses, and should be reported as recommended for other biological fluids^[64]^.

**Figure 3:**
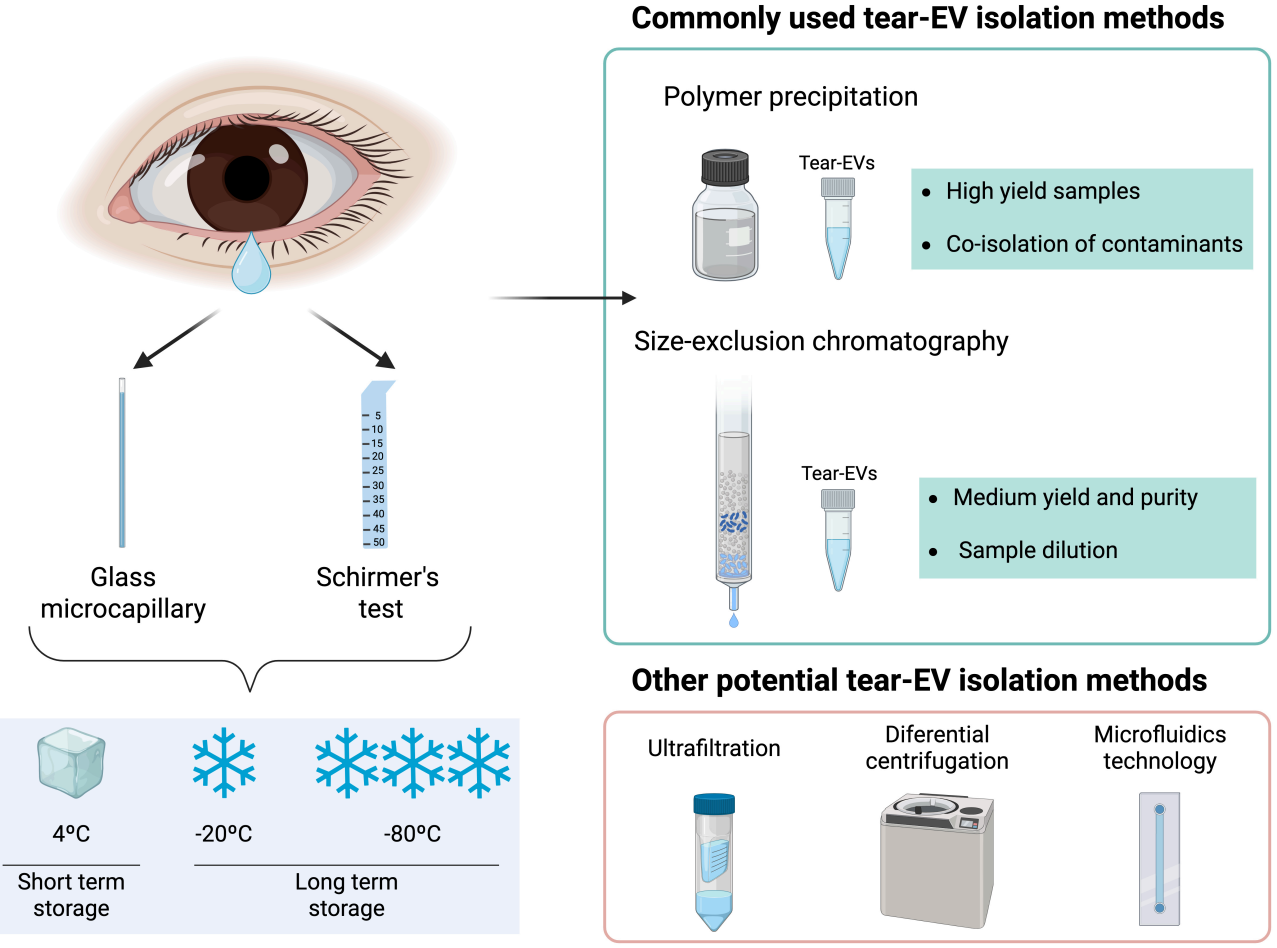
Schematic representation of methodologies for tear collection and EV isolation. Created in BioRender. Borràs, F. (2025) https://BioRender.com/cpfv79z. EV: Extracellular vesicle.

When using strips for tear sampling, it is important to avoid contact with the eyelid or the skin beneath the eye to minimize contamination by epithelial cells and their proteins, such as keratins^[65]^. Collected strips can be stored dry or submerged in a specific buffer (wet storage), such as phosphate-buffered saline or sodium chloride (NaCl), and maintained at room temperature, refrigerated (4 °C), or frozen (-20 and -80 °C) [Figure 3].

According to the literature, wet storage results in less variability in tear fluid protein concentration compared to dry storage, while storage at -20 or -80 °C is preferable to room temperature or 4 °C^[66]^. When tears are collected using a microcapillary tube, they are typically frozen directly at -80 °C until analysis^[61,62]^. Regarding storage duration, long-term storage (> 4 months at -70 °C) has been reported to reduce total tear protein concentration^[67]^, whereas a more recent study determined that there was no effect when tear samples were stored for up to 6 hours at 4 ºC before freezing at -80 ºC^[68]^, with frozen samples remaining stable for 6 months before analysis.

Collected tear volumes are often limited, depending on the patient’s condition and disease^[69]^. Therefore, for EV studies, it is crucial to adopt EV isolation techniques that maximize purity while minimizing yield loss. Numerous EV-isolation methods are available^[70]^, which can be classified into three groups based on sample purity and yield: high-yield but low-purity methods (ultrafiltration, polymer precipitation), intermediate methods balancing purity and yield (size exclusion chromatography, ultracentrifugation), and high-purity but low-yield methods (affinity isolation, microfluidics technology)^[70]^.

The selection of the EV isolation method should consider the intended purpose of the experiment, as different methods may influence the characteristics of isolated EVs^[71]^, potentially masking their function or hampering biomarker discovery.

Achieving an optimal balance between recovery and selective separation of EVs from co-isolates remains challenging, and the chosen method may differ depending on the source biological fluid and its composition. As for tear-derived EVs, most published studies employ polymer precipitation, with fewer using size exclusion chromatography, reflecting different experimental goals [Figure 3].

Polymer precipitation enables high-yield EV recovery through the use of hydrophilic polymers that decrease EV solubility, allowing for pellet formation^[72]^, although it usually co-isolates contaminants and undesired components, affecting the screening process^[73]^. Given that tear fluid contains relatively low levels of proteins and cellular debris compared to other biological fluids, polymer precipitation could be considered an optimal method for tear-EV isolation. Commercial kits based on this method are straightforward, rapid, and widely applicable for biomarker discovery. A variety of kits exist for fluids such as urine, blood, or plasma, but no commercial kit has been specifically designed for tear-EV isolation, and researchers typically adapt protocols from kits intended for other fluids^[74,75]^.

In contrast, size exclusion chromatography (SEC) achieves a balance between purity and yield by separating sample molecules based on size and weight^[76]^. Smaller molecules, such as free proteins, elute later than EVs, resulting in fractions enriched in different components. Although SEC achieves high purity, it may dilute EV samples, so starting volume and sample composition must be considered. To ensure sufficient volume and representative EV composition, many researchers pool tear samples from multiple donors for downstream analyses^[77,78]^. Regarding EV storage, standardized procedures are lacking, and studies report conflicting results. The most common method is storage at -80 °C, although repeated freeze-thaw cycles could affect EV functionality^[79]^. ISEV guidelines^[80]^ acknowledge the influence of preservation on EVs and encourage further research to clarify the effects of storage conditions on EV recovery and function. Thus, more advancement is required to address volume limitations and standardize tear-EV sample handling, storage, and processing.

## TEAR-DERIVED EVS AS POTENTIAL BIOMARKERS IN OCULAR DISEASE

Given their association with the ocular surface and proximity to disease sites, tears are considered a potential reservoir of protein, lipid, and molecular biomarkers for a variety of ocular conditions. Inflammatory eye diseases are among these pathologies, primarily encompassing degenerative conditions with inflammatory components^[44]^. The most prevalent of these diseases include glaucoma, dry eye syndrome, and diabetic retinopathy.

Glaucoma is a progressive optic neuropathy frequently associated with elevated intraocular pressure (IOP) resulting from impaired aqueous humor drainage. It affects the optic nerve and remains the leading cause of permanent blindness worldwide^[81]^.

Dry eye is a multifactorial disease of the ocular surface characterized by loss of homeostasis of the tear film and accompanied by ocular symptoms. Key etiological factors include tear film instability and hyperosmolarity, ocular surface inflammation and damage, and neurosensory abnormalities^[82]^.

Diabetic retinopathy, a common diabetes complication, arises from high blood sugar levels and is characterized by the growth of abnormal blood vessels in the retina. If inadequately controlled, complications of this pathology can lead to vision loss^[83]^.

Tear-derived EVs were first identified only a few years ago. Initial studies provided a detailed characterization of tear-EVs, confirming their nature and examining their morphological and molecular features^[84]^. This is an emerging and rapidly expanding field, with recent studies increasingly focusing on tear-EVs as a source of biomarkers for ocular diseases^[85]^ [Table 2].

**Table 2:** Summary of diseases and methodologies for biomarker profiling in tear samples, including sample number, collection method, EV isolation and characterization, and biomarker discovery

**Aim**	**Methodology**	**Disease**	**Sample number**	**Tear collection method**	**EV isolation method**	**EV characterization**	**Biomarker discovery**	**References**
Proteomic analysis	Liquid chromatography	Graves’ ophthalmopathy	48 patients 16 controls	Schirmer strips	Polymer precipitation	NTA, TEM, western blot, Luminex, ELISA	IL-1, IL-18, Caspase-3, C4A, APOA-IV	[75]
Proteomic analysis	Liquid chromatography	Sjögren syndrome	27 patients	Schirmer strips	Size exclusion chromatography	NTA, flow cytometry	CPNE1, CALM	[77]
Proteomic analysis	Liquid chromatography	Sjögren syndrome	25 patients 10 controls	Schirmer strips	Size exclusion chromatography	NTA, flow cytometry	STOM, ANXA4, ANXA11	[78]
Proteomic analysis	Liquid chromatography	Glaucoma	16 patients 17 controls	Schirmer strips	Instrumental cell sorting	Flow cytometry	PML	[86]
Transcriptomic analysis	RNA-sequencing	Dry eye disease	5 patients 5 controls	Washing ocular surface	Polymer precipitation	ELISA, TEM	miR-6506-5p, miR-6750-3p, miR-3669, miR-7853-5p, miR-4492	[87]
Transcriptomic analysis	RNA-sequencing	Dry eye disease	10 patients 2 groups	Schirmer strips	Polymer precipitation	NTA, TEM, flow cytometry, western blot	SCNM, mir-130b	[88]
Transcriptomic analysis	RNA-sequencing	Diabetic retinopathy	19 patients 11 controls	Schirmer strips	Negative-pressure	NTA, TEM, western blot	miR-145-5p, miR-214-3p, miR-218-5p, and miR-9-5p	[89]

EV: Extracellular vesicle; NTA: nanoparticle tracking analysis; TEM: transmission electron microscopy; ELISA: enzyme-linked immunosorbent assay.

In this sense, proteomic studies using liquid chromatography with tandem mass spectrometry analysis have been conducted to analyze tear-EV protein expression in diseases such as glaucoma, Graves’ ophthalmopathy, and primary Sjögren syndrome. Rossi *et al.* collected tears via the Schirmer test and isolated EVs using instrumental cell sorting based on specific EV markers. They reported PML protein activation in tear-derived EVs from glaucoma patients, which is involved in apoptosis and regulated, among others, by ACTG1, Actin, and lysozyme C^[86]^. Their analysis also revealed that EV cargo was enriched in lysozyme, one of the most expressed tear proteins, involved in the recruitment of neutrophils^[90]^. Regarding Graves’ ophthalmopathy, an autoimmune thyroid eye disease that affects the ocular surface, Shi *et al.* reported overexpression of IL-1 and IL-18 in tear EVs compared to healthy controls^[75]^. These cytokines are well-known mediators of inflammation and the aging process^[91]^. Tears were collected using the Schirmer test, and tear-EVs were subsequently isolated with a commercial kit based on precipitation. Aqrawi *et al.* studied tear-derived EVs from patients with primary Sjögren syndrome, an autoimmune disease affecting exocrine glands including salivary and lacrimal glands. They collected tears using the Schirmer test, and isolated EVs via size exclusion chromatography for proteomic analysis. They observed upregulated proteins in patients compared to healthy controls, involved in NF-α signaling and B cell survival, such as thioredoxin-dependent peroxide reductase (PRDX3), copine (CPNE1), and aconitate hydratase (ACO2)^[77]^. The authors also reported upregulated cellular processes related to retina homeostasis and central innate and adaptive immune responses in the patient group^[78]^.

Transcriptomic analyses of tear-derived EVs from dry eye disease and diabetic retinopathy patients have also been performed via RNA sequencing. Pucker *et al.* collected tears by eye wash with PBS and isolated EVs using polyethylene glycol polymer precipitation. RNA sequencing of EV microRNAs revealed several upregulated microRNAs involved in inflammation (miR-127-5p, miR-1273h-3p, miR-1288-5p, miR-130b-5p, miR-139-3p, miR-1910-5p, miR-203b-5p, miR-22-5p, and miR-4632-3p)^[87]^. Similarly, Cross *et al.* analyzed tear-EV RNA profiles from two groups of dry eye patients, identifying differential expression of sodium channel modifier 1 (SCNM1) and immature miRNA-130b^[88]^. In this case, tears were collected via the Schirmer test, and EVs were isolated using a commercial precipitation kit. Finally, Hu *et al.*^[89]^ studied tear-EV RNA expression in patients with diabetic retinopathy. Tears were collected by the Schirmer test, and EVs were isolated using a custom harmonic oscillation-based kit. They identified several dysregulated microRNAs, including miR-145-5p, related to NF-kB signaling and endothelial dysfunction^[92]^; miR-9-5p, implicated in angiogenesis and insulin secretion^[93]^; and miR-214-3p and miR-218-5p, strongly associated with insulin resistance^[94]^.

These findings highlight the potential of tear-derived EVs in the diagnosis of inflammatory and autoimmune eye diseases. However, the use of tear EVs for biomarker discovery remains in its initial stages, and further research is needed to establish their clinical utility.

## TEAR-EV POTENTIAL IN NEURODEGENERATIVE DISEASE BIOMARKER DISCOVERY

Tear fluid provides specific information about the tissues beneath the eye. However, systemic diseases can also influence tear proteome patterns. Consequently, analysis of tear film protein composition has emerged as a useful diagnostic approach not only for ocular conditions but also for systemic diseases^[56,95]^. Because the retina is an extension of the central nervous system^[96]^, the brain-eye axis has attracted increasing attention in the study of neurodegenerative disorders such as multiple sclerosis, Alzheimer’s disease, and Parkinson’s disease. Given that tears are a more accessible and less complex body fluid than serum or plasma and their collection is much less invasive^[95]^ than that of aqueous or vitreous humor, there is a growing interest in their study as a source of biomarkers for neurodegenerative diseases. In particular, several studies have explored tear-derived EVs as promising biomarker candidates.

Pieragostino *et al.* found microglia- and neural-derived EVs in tears through multiple proteomic studies in patients with multiple sclerosis. The authors collected CSF and tears using lumbar puncture and Schirmer test, respectively, and isolated EVs from both fluids by cell sorting based on specific markers. Their results showed that EVs from the CSF and tears of multiple sclerosis patients shared similar protein profiles, with a 73.1% overlap, particularly in proteins involved in inflammation, angiogenesis, and immune response signaling^[97]^. In another study, Salvisberg *et al.* reported reduced levels of proline-rich protein 4 in tears, CSF, and serum of patients with multiple sclerosis^[98]^. Proline-rich protein 4, a major tear protein, has previously been associated with various ocular diseases including dry eye syndrome^[31,99]^ and glaucoma^[90]^. These findings suggest that the protein cargo of tears, particularly within tear EVs, may reflect the pathophysiological state of the CSF.

Other investigations focused on the close relationship between the brain and the eye have explored the implications of tear fluid in Alzheimer’s disease. Lee *et al.* performed proteomic analyses and identified adenylyl cyclase-associated protein 1 as a potential tear biomarker for the disease^[100]^. This protein is essential for the proper functioning of cones, and altered levels may contribute to synaptic dysfunction^[101]^. This finding is consistent with another study reporting differential expression of adenylyl cyclase-associated protein 1 in serum-derived EVs from Alzheimer’s patients^[102]^, reinforcing the predictive value of tear biomarkers in this neurodegenerative disease. Following this line, a pilot study in Parkinson’s disease revealed some disease-specific protein patterns in patients’ tear fluid. The authors found 21 proteins significantly increased and 9 downregulated compared to controls, most of them involved in immune response, lipid metabolism, and oxidative stress^[103]^. Notably, in line with the aforementioned study on multiple sclerosis^[98]^, proline-rich protein 4 was among the downregulated proteins. These shared alterations across different biofluids point to molecular crosstalk among CSF, plasma, and tears, underscoring the diagnostic potential of tear-derived EVs in neurodegenerative disorders.

## ADVANTAGES, LIMITATIONS, AND FUTURE PERSPECTIVES

As outlined throughout this review, the use of tears as a source of biomarkers has gained considerable attention in recent years, primarily due to their easy accessibility, non-invasive collection, and simple sampling procedures. Numerous studies have explored the potential of this biofluid to reveal key molecular pathways involved in the progression of various diseases^[95]^. These include ocular conditions such as dry eye disease, diabetic retinopathy, and glaucoma^[49]^. Tears are particularly valuable in this context, as they originate close to the site of pathology and specifically reflect the (patho)physiological state of the eye. They can mirror changes in inflammatory and pro-fibrotic markers induced by topical medications prescribed for diverse inflammatory eye diseases^[104]^. Recent reviews have summarized the key molecular mechanisms underlying glaucoma development^[105]^, offering new perspectives for preventing ocular fibrosis in clinical practice^[106]^. On the other hand, tears can also serve as a valuable tool for detecting systemic disorders, including neurodegenerative diseases and certain cancers^[107,108]^. For instance, breast cancer-specific miR-21 and miR-200c have been identified in tear EVs from patients with metastatic disease, pointing to the broader diagnostic potential of tear-derived EVs^[50]^.

The potential of tear-based biomarkers in different pathological contexts has been reviewed previously^[109]^. However, research on tear-EV-derived biomarkers for ocular, neurodegenerative diseases, and cancer is still scarce. Recent reviews have highlighted the diagnostic potential of EVs derived from different ocular fluids, including aqueous humor, vitreous humor, and tears^[16,110,111]^. In fact, although the collection of aqueous^[112,113]^ and vitreous humor^[114,115]^ is more invasive, these fluids may still have specific applications in ocular disease research. Nevertheless, the non-invasive collection of tear-EVs offers a clear advantage, providing a rapid, simple, and minimally invasive method for identifying biomarkers for early disease detection and monitoring.

Despite these advantages, several technical limitations must be considered. The first challenge lies in the method of tear collection. Currently, the most widely used techniques - microcapillary tubes and Schirmer strips - are both minimally invasive and easy to implement. However, microcapillary collection can be hindered by involuntary blinking or low tear volume^[3]^, while the application of anesthesia may have an impact on tear composition. When performed with topical anesthesia, the Schirmer test measures basal tear production under unstimulated conditions; without anesthesia, however, it induces irritation and reflex tearing, thus measuring both basal and reflex tear secretion^[69]^. Consequently, the collection method itself may influence tear composition. For example, two of the studies discussed in this review, which conducted transcriptomic analyses of tear-derived EVs in patients with dry eye syndrome^[87,88]^, despite using the same EV isolation methodology, employed different tear collection techniques and reported distinct results. While both studies included a limited number of patients, the findings suggest that the sampling method may significantly affect outcomes. Further comparative analyses are therefore warranted to clarify the extent of these differences^[88,116]^.

Once collected, an equally critical step is the selection of an appropriate EV isolation method. Size exclusion chromatography, either alone or combined with ultrafiltration, or ultracentrifugation is commonly considered a gold-standard approach in EV research^[80]^. However, the limited yield obtained from tear samples represents a significant bottleneck in tear-EV analysis. Notably, tears contain significantly less protein than serum or plasma, which may facilitate cleaner EV isolation. On the other hand, SEC may further dilute samples, resulting in the loss of valuable EV fractions. To address this, some researchers pool tear samples from multiple individuals before applying SEC^[77,78]^. This approach increases sample volume and reduces inter‐ or intra‐subject background noise^[36]^, but at the cost of sample identity and potentially meaningful individual variability. Alternatively, commercial precipitation-based kits are frequently used because they provide higher yields, although with reduced purity^[75,88,89]^. As with other EV studies, the choice of isolation method should ultimately depend on the specific objectives of the research.

Another source of variability across studies arises from storage conditions for tear samples and/or isolated EVs. Most researchers store tear samples at 4 °C for short-term use, followed by freezing at -80 °C for long-term storage, either before or after EV isolation. However, standardized protocols for EV storage in low-volume fluids such as tears are lacking, and key details including temperature, storage duration, and buffer composition are often omitted from published studies. Establishing methodological standards and reporting all preanalytical details will be critical to improving reproducibility in future research.

## CONCLUSIONS

Significant progress has been made in recent years in exploiting tear-EV characteristics as biomarkers. Nonetheless, further efforts are required to standardize and fully report tear-EV collection and isolation methods to ensure reproducibility across laboratories. In this context, the adoption of Standard PREanalytical Code (SPREC) parameters^[117]^ for codifying and documenting preanalytical conditions in biofluid specimens may help in assessing EV parameters relevant to clinical practice. Together with adherence to ISEV guidelines for reporting EV research^[39]^, these measures will advance tear-derived EV research as a promising frontier in predictive and diagnostic biomarker discovery, particularly for ocular and neurodegenerative diseases.

## DECLARATIONS

### Copyright

© The Author(s) 2025.
